# A New Approach to Identifying the Drivers of Regulation Compliance Using Multivariate Behavioural Models

**DOI:** 10.1371/journal.pone.0163868

**Published:** 2016-10-11

**Authors:** Alyssa S. Thomas, Taciano L. Milfont, Michael C. Gavin

**Affiliations:** 1 School of Geography, Environment and Earth Sciences, Victoria University of Wellington, Wellington, New Zealand; 2 School of Psychology, Victoria University of Wellington, Wellington, New Zealand; 3 Human Dimensions of Natural Resources, Colorado State University, Fort Collins, Colorado, United States of America; Charles University, CZECH REPUBLIC

## Abstract

Non-compliance with fishing regulations can undermine management effectiveness. Previous bivariate approaches were unable to untangle the complex mix of factors that may influence fishers’ compliance decisions, including enforcement, moral norms, perceived legitimacy of regulations and the behaviour of others. We compared seven multivariate behavioural models of fisher compliance decisions using structural equation modeling. An online survey of over 300 recreational fishers tested the ability of each model to best predict their compliance with two fishing regulations (daily and size limits). The best fitting model for both regulations was composed solely of psycho-social factors, with social norms having the greatest influence on fishers’ compliance behaviour. Fishers’ attitude also directly affected compliance with size limit, but to a lesser extent. On the basis of these findings, we suggest behavioural interventions to target social norms instead of increasing enforcement for the focal regulations in the recreational blue cod fishery in the Marlborough Sounds, New Zealand. These interventions could include articles in local newspapers and fishing magazines highlighting the extent of regulation compliance as well as using respected local fishers to emphasize the benefits of compliance through public meetings or letters to the editor. Our methodological approach can be broadly applied by natural resource managers as an effective tool to identify drivers of compliance that can then guide the design of interventions to decrease illegal resource use.

## Introduction

One of the leading causes of failures in fisheries management is non-compliance with regulations [1]. Fishers’ rule-breaking can undermine regulation efficiency, cost effectiveness and the legitimacy of the management regime [2,3] and compromise conservation goals [4]. However, in order to effectively address non-compliance, managers must first understand the primary drivers of rule-breaking behaviour [5,6]. Although compliance decisions are motivated by a complex mix of factors, including enforcement, moral norms, legitimacy and the behaviour of others [7,8], the majority of previous studies have only explored the effects of different factors *separately*, rather than how well models encompassing distinct factors *simultaneously* explain behaviour. Our research used a New Zealand recreational fishery to test the effectiveness of comprehensive behavioural models in predicting fishers’ compliance with two different regulations.

Although Oh et al. [9] used structural equation modeling to examine site substitution in a fishery, the current study appears to be the first to use structural equation modeling in fisheries research or natural resource management to test multiple behavioural models to explain regulation compliance. To our knowledge this study is also the first test of psycho-social models, such as the Theory of Planned Behaviour [10], in fisheries management. Although the tested models explained a fisher’s compliance decision with varying degrees of success, the best fitting models explained over 55% and 35% of the variance for the size and daily limit, respectively. Results demonstrate the usefulness of using comprehensive behavioural models to examine compliance behavior, including psycho-social ones.

### Theoretical framework

There have been multiple attempts (e.g., [11–13]) at understanding both regulation compliance and pro-environmental behaviour using a series of key behavioural drivers. Although non-compliance was originally viewed as a calculated decision based on risk evaluation (instrumental model; see [14–16]), more recent studies have argued that external influences on individuals are often more prevailing (normative model; see [5, 17–18]). The instrumental model assumes that individuals are motivated solely by self-interest and that compliance is the result of external contingencies [14]. Alternatively, the normative model focuses on the intrinsic motivations of the individual and suggests that compliance behaviour is the result of an individual’s morality and internalized norms. Thus, compliance is achieved when people consider the regulations to be fair, legitimate and meaningful (e.g.,[19–20]).

We tested seven different models combining instrumental and normative perspectives and inclusive of 16 factors. The models are summarised in Table 1 and described in more detail below. In brief, the seven models included three compliance models from the literature (models #s 1, 2 and 4 in Table 1), a combination of the two most basic compliance models (# 3), a model of pro-environmental behavior (# 5) and a slightly modified version (# 6), and an all-inclusive model (# 7) that allowed the addition of potentially important factors that were not part of established behavioural models.

**Table 1:** Overview of seven behavioural models of fisher compliance decisions.

*Model*	*Factors*
1. Instrumental [14]	probability of detection, probability of conviction and penalty if convicted
2. Theory of Planned Behaviour [10]	attitude, social norm (descriptive and injunctive) and perceived behavioural control
3. Instrumental and Theory of Planned Behaviour combined [10, 14]	Probability of detection, probability of conviction, penalty if convicted, attitude, social norm (descriptive and injunctive) and perceived behavioural control
4. Legitimacy [3, 19]	meaningful rule, involvement in decision-making process and outcome fairness and effectiveness
5. Bamberg and Möser [21]	factors from the Theory of Planned Behaviour *plus* problem awareness, attribution, guilt and moral norm
6. Modified Bamberg and Möser [10,21]	factors from the Bamberg and Möser model *plus* a direct path from social norm to compliance, based on the Theory of Planned Behaviour
7. Fully Inclusive [3, 10, 14, 19, 21]	factors from the modified Bamberg and Möser model plus probability of detection, probability of conviction, penalty if convicted, meaningful rule, involvement in decision-making process, outcome fairness and effectiveness, and regulation knowledge

An instrumental view of compliance, based on Becker [14], was the first model tested. This model presents non-compliance as the outcome of the expected net gain from breaking the law, the risk of detection, the probability of conviction, and the severity of punishment [22]. If the expected illegal gain is greater than the three combined risks, the individual is predicted to violate. The second alternative model tested was the Theory of Planned Behaviour [10], a well-known framework for modeling behaviour [23]. According to this model, behavioural intentions are the most proximal determinant of behaviour, and an individual’s intention to perform a particular behaviour is in turn predicted by three socio-cognitive factors: attitudes, subjective norms, and perceived behavioural control (an individual’s estimate of their ability to perform a specified behaviour). However, intention was not included as a factor and the study modelled behaviour directly. The six variables included in these two models were combined into a third model representing both the instrumental and basic normative views of compliance.

Four normative factors (meaningful rule, involvement in decision-making process, and outcome fairness and effectiveness) were used as measures of the management regime’s legitimacy and tested together as the fourth model. Outcome fairness refers to who gets more of the resource under the regulation, and outcome efficiency refers to how well the regulation objectives are achieved [19]. Although procedural fairness and effectiveness are included in Tyler’s [19] model of legitimacy, fishers interviewed in a pilot study did not mention these factors, nor did any news articles related to the particular fishery, so they were not included in the model.

**Fig 1:**
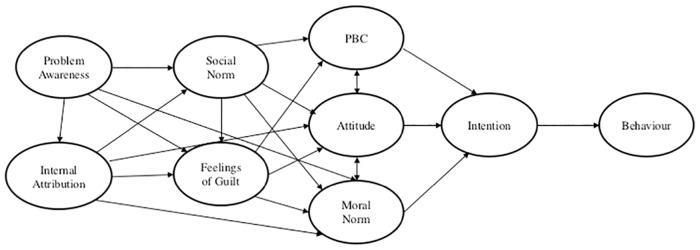
Model of pro-environmental behaviour from Bamberg and Möser [21].

The fifth model we tested was Bamberg and Möser’s [21] framework of pro-environmental behaviour. Bamberg and Möser conducted a meta-analysis of 46 studies and proposed eight psycho-social factors that influence pro-environmental behaviour: the factors from the Theory of Planned Behaviour plus problem awareness, attribution, guilt, and moral norm (Fig 1). Our sixth model, was a modified version of Bamberg and Möser’s framework. Whereas in the original Bamberg and Möser model the social norm influenced an individual’s behaviour indirectly, the modified version added a direct path from the social norm to compliance because previous studies [23–24] have demonstrated that social norms can directly influence behaviour. Finally, our seventh model contained all 15 variables from the other six models and one new variable, regulation knowledge. Other compliance research (e.g., [5, 25]) strongly supports this inclusion, as without correct regulation knowledge non-compliance may be widespread.

The social norm contained in five of these models is an injunctive social norm, or an individual’s perception of what others believe to be the appropriate conduct. Yet a descriptive social norm, or what an individual believes others are doing, can also have a very strong influence on an individual’s behaviour [26]. Thus, injunctive and descriptive social norms were measured separately and included in the relevant models as two factors determining the overall social norm.

## Materials and Methods

### Study area

**Fig 2:**
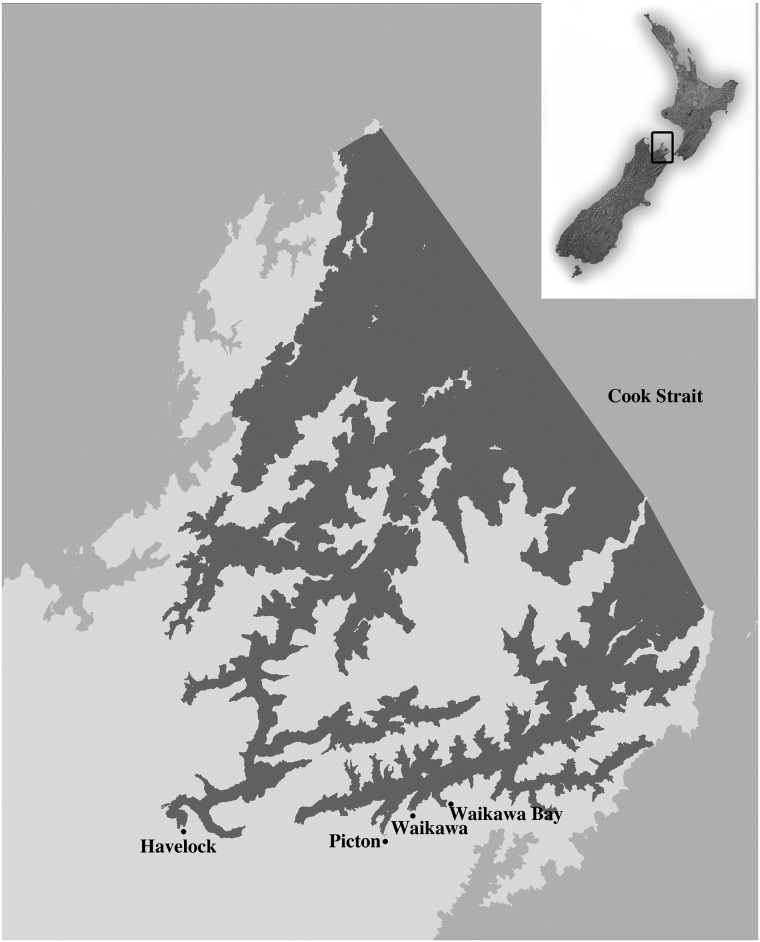
Location of the Marlborough Sounds, New Zealand [29]. The boundaries of the Marlborough Sound’s recreational blue cod fishery are indicated by shading and the study sites are also shown. New Zealand map sourced from LINZ (Crown Copyright Reserved) and Marlborough Sounds map modified from the Ministry of Primary Industries [30].

We examined the recreational blue cod fishery in the Marlborough Sounds, South Island, New Zealand (Fig 2). The warm climate, sheltered and accessible nature of the Marlborough Sounds, coupled with excellent fishing opportunities, makes it a popular holiday destination. The recreational fishing pressure can thus be intense; with blue cod (*Parapercis colias*) the most frequently caught species in the South Island [27]. In April 2011 the fishery was re-opened after a 3.5-year closure with a complex suite of regulations including a daily limit of two blue cod per person per day and minimum and maximum size requirements (between 30 and 35cm). Previous analysis by the authors estimated that in 2012 the size limit was violated by over 40% of fishers and the daily limit by at least 10% [28]; underling the need for information on the drivers of these behaviours.

### Data collection

The initial contact with most survey participants was through face-to-face intercept surveys carried out over six weeks in January-February 2013 (summer) at four boat launch locations in the Marlborough Sounds. Recruitment also took place via email to the fishers previously interviewed, plus contacts via newspaper, radio, magazine and flyers in order to enlarge the sample size and minimise sampling biases. Informed consent was used, with the first page asking for participants’ agreement to take part in the research. Assurance was provided that all responses would remain confidential, could not be traced back to any individual and were therefore anonymous. The survey was carried out online using the survey platform Qualtrics (www.qualtrics.com). Online surveys provide a greater feeling of anonymity which can increase honest responses to direct questions [31].

It is important to note potential biases with the sampling protocol used. Fishers who did not use one of the boat ramps surveyed, or used the ramps on a different day, had less of a chance of taking part in the survey. The sample was also not strictly random so results may be biased towards more interested respondents. Since the survey was online, fishers with less access to or familiarity with computers may be under-represented in the sample population.

The survey started with several general questions on fishing in the Marlborough Sounds (not included) followed by three questions asking for the correct daily limit, minimum and maximum sizes for blue cod to test fisher knowledge of regulations. The next three sections focused on the potential drivers of compliance with both the recreational daily and size limits for blue cod. We measured all but one of the factors (probability of conviction) using three or four statements, and at least one statement per factor was reverse scored to minimize bias (please refer to S1 Table). For the majority of statements agreement was measured using a Likert-type scale anchored by 1 (strongly disagree) and 5 (strongly agree). The probability of conviction was assessed using a single question with five choices ranging from very high to very low. Finally, towards the end of the survey participants were directly asked whether they had violated either regulation in 2012.

Concerns exist regarding the use of direct questioning to gather information on illegal resource use due to response biases driven by fear of retribution [32]. In this case we have chosen to use data from direct questioning for three main reasons. First, the online survey format we used allows for greater anonymity and more honest answering [31]. Second, in a prior study in the same region [30], we found no significant differences in estimates of non-compliance with the daily limit based on direct versus indirect questioning approaches. Third, the use of direct questioning allowed us to link responses to questions on non-compliance with data on potential drivers of behavior from the same respondents, permitting statistical modeling with more variables in each model and lower sample sizes. We also emphasize that regardless of the data collection approach, some respondents will invariably respond dishonestly or refuse to answer [33], meaning errors will exist in any estimates of drivers of non-compliance.

### Data analysis

Prior to testing the full models, the 16 constructs were tested separately for validity (confirmatory factor analysis) and reliability (Cronbach’s alpha) to determine the best-fitting measurement models using SPSS and AMOS. Structural equation modeling was carried out in MPlus with WLSMV (weighted least square means and variance-adjusted) used as the estimator. This approach provides the best results for modeling categorical data [34]. For all tested models, except the fully inclusive (model 7), non-significant paths and factors were removed but no additional paths were added.

## Results

The final sample consisted of 320 fishers, 82% male and 45% from the Marlborough region. Responding fishers ranged from 22 to 88 years in age (*M* = 54, *SD* = 12), with one to 70 years (*M* = 28, *SD* = 17) of local fishing experience. Eighty-nine percent of respondents owned a boat and 37% owned a holiday home in the Sounds. As no permits are needed for saltwater fishing in New Zealand, current demographic information is not easily obtained. The most recent demographic information, aside from this study, is from a 2009 project [35] which found similar results to those above and estimated the number of fishers at 9,100. However, this estimate is likely to have significantly decreased as conversations with fishers over the course of the research revealed that the new regulations had resulted in many fishers going elsewhere in New Zealand where there are higher limits. For the questions testing regulation knowledge, 91% of fishers chose the correct daily limit (two), 89% the correct minimum size (30cm) and 94% the correct maximum size (35cm). Structural equation modeling results are presented in Table 2.

**Table 2:** Model fit indices for models tested to explain fisher compliance with regulations.

Model	Model Fit Indices							
	*Regulation*	*x*^*2*^	*df*	*x*^*2*^*/df*	*RMSEA*	*CFI*	*TLI*	*R*^*2*^
1. Instrumental								
	Daily Limit	2.55	1	2.55	0.03 (0.00–0.12)	0.92	0.88	0.08
	Size Limit	0.12	1	0.12	0.00 (0.00–0.02)	1	2.39	0.03
2. Theory of Planned Behaviour								
	Daily Limit	58.97	5	11.79	0.19 (0.14–0.23)	0.55	0.19	0.39
	Size Limit	142.97	4	35.64	0.33 (0.29–0.38)	0.46	-0.21	0.5
3. Instrumental + Theory of Planned Behaviour								
	Daily Limit	98.27	13	7.56	0.14 (0.12–0.17)	0.43	0.2	0.44
	Size Limit	218.82	13	16.83	0.22 (0.20–0.25)	0.33	0.08	0.52
4. Legitimacy								
	Daily Limit	16.01	7	2.29	0.06 (0.02–0.11)	0.72	0.6	0.11
	Size Limit	10.8	5	2.16	0.06 (0.00–0.11)	0.97	0.95	0.09
5. Bamberg & Möser								
	Daily Limit	66.36	22	3.02	0.08 (0.06–0.10)	0.91	0.86	0.29
	Size Limit	55.41	15	3.7	0.09 (0.07–0.12)	0.95	0.91	0.55
6. Modified Bamberg & Möser								
	Daily Limit	2.1	1	2.1	0.06 (0.00–0.17)	0.99	0.96	0.37
	Size Limit	12.62	6	2.1	0.06 (0.00–0.10)	0.99	0.96	0.56
7. Full Inclusive								
	Daily Limit	143.48	48	2.99	0.08 (0.06–0.10)	0.83	0.76	0.29
	Size Limit	162.63	54	3.01	0.08 (0.07–0.09)	0.91	0.87	0.56

X^2^/df = the ratio of chi-square to degrees of freedom; RMSEA = root mean square error of approximation; 90%CI = 90 percent confidence interval; CFI = comparative fit index; TLI = Tucker-Lewis fit index. Shading indicates the best-fitting models.

The reported measures of model fit and the r-squared value were used to determine the model that best explained the data. When assessing model fit, both absolute and relative measures of model fit were used with the following cut-offs: chi-squared test/degrees of freedom (x^2^/df) ≤ 3, root mean square error of approximation (RMSEA) ≤ 0.06 (both absolute fit indices), and comparative fix index (CFI, a measure of relative fit) ≥ 0.95. A non-normalized measure of model fit was also used, the Tucker-Lewis Fit Index (TLI), as it includes a penalty for overly complex models. The TLI is interpreted in the same way as CFI where a good model fit value is ≥ 0.95 [34].

Comparing the seven competing models for the size limit, three had comparable good fit based solely on the CFI: instrumental, legitimacy and the modified Bamberg and Möser. However, closer examination of the fit statistics showed that the modified Bamberg and Möser model had the overall best fit, with the highest CFI and TLI of any of the tested models. Importantly, this model also explained more than five times the variance of the legitimacy-based model (56% vs. 9%) and can be regarded as the best-fitting model.

**Fig 3:**
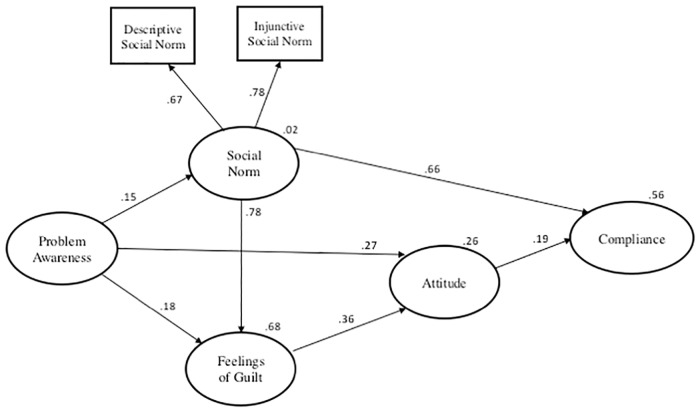
Graphical output of the selected best-fitting model for fishers’ compliance with the size limit. Numerical values on arrows are standardized regression coefficients (*β*) and values in the top right of ovals representing the constructs are coefficients of determination (R^2^).

The results from the modified Bamberg and Möser model (Fig 3) demonstrate that social norms had the largest influence (*β* = 0.66) on a fisher’s decision to comply with the size limit. As the social norm increased, the probability of compliance also increased. In other words, as the social pressure to comply became more prominent, either through more fishers complying or disapproving of violators, a fisher was more likely to comply with the regulation themselves.

Specifically, injunctive norms (*β* = 0.78) had a stronger contribution to the overall social norm construct compared with descriptive norms (*β* = 0.67). A fisher’s belief as to whether other fishers would approve of keeping blue cod outside the size limit had a larger influence than their perception of whether the majority of fishers complied with the size limit. Additionally, a fisher’s attitude had a smaller (*β* = 0.19) direct influence, with a more positive attitude increasing the probability of compliance. This model was better fitting (*x*^*2*^(2) = 47.51, *p* = 0.000) then an alternative model where the paths for both social norm and attitude were fixed to be equal, confirming that social norms have a stronger influence on fishers’ compliance than attitude. Moreover, both problem awareness and feelings of guilt indirectly influenced compliance behaviour via attitude.

**Fig 4:**
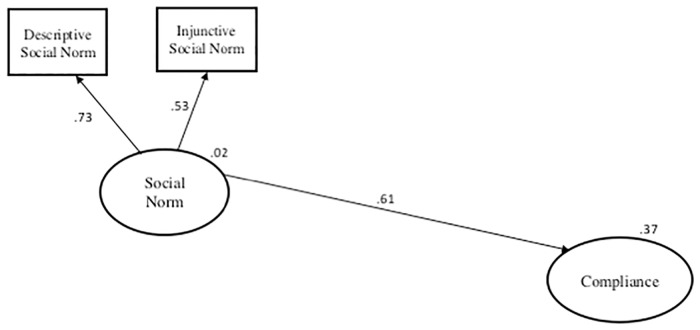
Graphical output of the selected best-fitting model for fisher’s compliance with the daily limit. Numerical values on arrows are standardized regression coefficients (*β*) and values in the top right of ovals representing the constructs are coefficients of determination (R^2^).

For the daily limit regulation, the modified Bamberg and Möser model was again the best fitting model, explaining four times the amount of variance (37% vs. 8%) as the next best-fitting model (instrumental). Therefore, the modified Bamberg and Möser was selected as the best determinant of a fisher’s decision to comply with the daily limit. Under this model (Fig 4) social norms were the only driver (*β* = 0.61) of daily limit compliance. In contrast to the size limit regulation, descriptive norms (*β* = 0.73) had a stronger contribution to the overall social norm construct compared with injunctive norms (*β* = 0.53). A fisher’s perception of whether the majority of fishers comply with the daily limit had a larger influence than their belief as to whether other fishers would approve of keeping within the daily limit. A stronger social norm increased the probability of a fisher complying with the regulation. All other variables were removed due to a lack of significant paths.

## Discussion

As resources to enforce regulations are often limited [36], it is critical to increase voluntary compliance through a greater understanding of the drivers of compliance. However, numerous potential drivers of compliance exist, and each driver is best addressed by a unique management intervention. In turn, the challenge becomes identifying the relative influence of different drivers of compliance. Here we present an approach to compare the relative explanatory power of different models of compliance. This method provides a robust mechanism for identifying drivers of behavior, which can lead to more targeted management interventions [37].

In the current study, the best-fitting compliance models contained no instrumental factors and showed that the overriding influence on fishers’ compliance with both the size and daily limits was social norms. This finding contrasts with compliance in many commercial fisheries that tends to emphasise instrumental factors [3, 22]. However, social norms have been found to be influential in other areas of natural resource management such as greening programs in China [38] and tree planting in Pakistan [39]. Based on the absence of instrumental factors in the selected models, a management strategy aiming to promote voluntary acceptance of these regulations, rather than increasing enforcement efforts, would be a more effective approach.

Once the drivers of compliance behavior are identified, targeted and timely behavioural interventions can be used to address regulation non-compliance and/or promote compliance behaviour. In the fishery we studied, such behavioural interventions should primarily focus on the social norms, with descriptive and injunctive norms addressed in different ways. The descriptive social norm is best targeted in a positive way by emphasizing that a majority of the fishers ‘do the right thing’ and comply with the size and daily limits; as focusing on the undesirable behaviour is likely to backfire [26]. Short articles in the local newspaper and fishing publications would be a straightforward approach to targeting the descriptive social norm.

For the injunctive social norm, we suggest using a ‘block leaders’ approach. This method uses volunteers to disseminate information, as fishers may be more likely to change their behaviour in response to social pressure from other fishers. Block leaders are “volunteers who help inform other people about a certain issue” [40] (pp.1774). In this case, block leaders are recreational fishers who are residents of the Marlborough area; ideally more experienced ones as they should have a greater influence. Stern [41] showed that norms are more important if there is a moral obligation attached to the desired action; so the block leaders could emphasize how compliance with the regulations is the best course of action as there is a collective responsibility to sustain the fishery for the future (a goal previously expressed by many fishers). Although we are unaware of any examples of an adaptive management approach in fisheries, Cialdini [42] provided an example of changing the social norm to promote pro-environmental behaviour. Heard et al. [43] also suggested changing social norms to bring about the desired behavioural changes in the catch and release of sharks.

In the present study, the best-fitting models explained more than half of the variance in an individual’s compliance decision with the size limit (56%) and more than a third (37%) of it for the daily limit. Although this result is strong considering the subject is an illegal and potentially sensitive behaviour, there is still a large proportion of the variance unaccounted for and suggests the need for an adaptive management strategy to reduce uncertainty. Under an adaptive management approach, structural equation modeling could be used to identify the primary drivers and then design targeted behavioural interventions in accordance with the findings. For example, in addition to letters to the editor written by block leaders, another way to address the descriptive social norm is the Ministry for Primary Industries providing a press release. It would be widely distributed through different media, highlighting high levels of compliance in helping the fishery to recover.

The use of control and experimental groups would provide a means to test the effectiveness of such an intervention in changing the descriptive social norm. Fishers in both groups could be asked about what they believe other fishers do, and then expose one group to press releases and or/articles on the extent of compliance. After exposure to this information, fishers would be asked the same questions on other fishers’ behaviour to test for any changes. As undesirable behaviours are often overestimated [44], changing fishers’ perceptions of this action could be effective in boosting the descriptive norm.

After implementation, managers should monitor the success of the interventions and evaluate if they have succeeded in increasing compliance. Based on the results, the interventions can be adjusted or re-evaluated as necessary. By providing managers with the information needed to design and implement cost-effective behavioural interventions, structural equation modeling allows managers to choose the best option and tailor their response to drivers specific to their area. A key advantage of this approach is providing information on relationships between the drivers, including both direct and indirect effects, for a more complete understanding.

## Ethics statement

The study was approved by the ethics committee of Victoria University of Wellington (#19097). Informed consent was used in this study, with the first page of the survey requiring participants’ agreement to take part in the research before proceeding to the next survey page and first question.

## Supporting Information

S1 Dataset Dataset. This includes the coding used for the constructs measured.
(CSV)

S1 Table Measures used to predict compliance with blue cod fishing regulations. (DOCX)
