# Supplementary material for: A New Approach to Identifying the Drivers of Regulation Compliance Using Multivariate Behavioural Models
Source: PLoS One. 2016 Oct 11;11(10):e0163868. doi: 10.1371/journal.pone.0163868 (PMC5058501; doi:10.1371/journal.pone.0163868)
Supplement: S1 Table — (DOCX) [file pone.0163868.s002.docx]

| **S1 Table.** **Measures used to predict compliance with blue cod fishing regulations.** | |
| --- | --- |
| **Construct** | **Items** |
| ***Meaningful Rule***  Whether an individual feels a particular rule is meaningful to them [1] | 1. There is valid science behind the blue cod regulations* 2. It is wrong to have to throw back a fish that is severely injured 3. MFish has the right to impose regulations governing blue cod fishing* 4. It is difficult to respect the blue cod regulations |
| ***Involvement in Decision-Making Process***  The degree to which an individual feels they were involved in making the rule in question [2] | 1. Recreational fishers were adequately involved in forming the blue cod regulations 2. Recreational fishers were well- represented in developing the blue cod management plans 3. My views were well-represented in developing the blue cod management plans 4. MFish did not listen to recreational fishers when developing the blue cod management plans* |
| ***Problem Awareness***  Awareness of and knowledge about environmental problems [3] | 1. The blue cod population was declining before the ban 2. It was necessary to do something about the overfishing of blue cod in the Sounds 3. The Sounds blue cod population needs to be carefully managed 4. The number of recreational fishers in the Marlborough Sounds has been increasing |
| ***Attribution***  An individual's belief that their behaviour is causing or contributing to the problem [3] | 1. My fishing practices did not contribute to the need for the blue cod regulations* 2. Going fishing in the Sounds puts pressure on the blue cod population 3. The blue cod regulations were needed because of recreational fishers 4. By following proper handling practices, my fishing does not harm the blue cod population* (deleted) |
| ***Probability of Detection***  The individual's perceived view on how likely they are to get caught performing the behavior in question [4] | 1. On a normal fishing trip I do not expect to see a MFish boat* 2. Enforcement by MFish is too focused on holiday periods* (deleted) 3. Enforcement by MFish is frequent enough to deter most fishers from violating the blue cod regulations 4. If you were to take more than the daily limit of blue cod, what would you consider to be the overall chance of being caught? |
| ***Probability of Conviction***  An individual’s assessment of the probability they will be convicted of the offense, if caught [4] | 1. If caught keeping more than the daily limit how likely is it that you would be penalised? |
| ***Penalty if Convicted***  What penalty an individual expects if they are convicted of rule-breaking [4] | 1. Potential penalties are strict enough to deter most recreational fishers form exceeding the daily limit for blue cod 2. I do not exceed the daily limit for blue cod because of the potential penalty 3. The penalty for taking more than the daily limit for blue cod is a concern to me 4. What penalty would you expect for exceeding the daily limit for blue cod (deleted) |
| ***Regulation Knowledge***  Knowledge and understanding of the applicable rule(s) governing the behaviour | 1. I have been informed of the daily limit for blue cod 2. I have easy access to information about the daily limit for blue cod 3. It is easy to understand what is required by the daily limit for blue cod 4. The daily limit for blue cod in the Marlborough Sounds (during the open season) is ___ (number) per person |
| ***Outcome Fairness***  Who gets more of the resource under the regulation [5] | 1. Marlborough-based recreational fishers are unfairly impacted by the daily limit* 2. The daily limit for blue cod has affected my fishing habits more than other people’s habits* (deleted for size limit) 3. Daily limits for blue cod are fairly applied to everyone 4. The daily limit for blue cod has a fair and equitable impact on recreational fishers |
| ***Outcome Effectiveness***  How well the regulation objectives are achieved [5] | 1. The daily limit is effective in leaving enough blue cod for the future 2. High levels of dissatisfaction among recreational fishers result from the daily limit for blue cod* (deleted for size limit) 3. Blue cod fish stocks are effectively conserved by the daily limit 4. A daily limit is not an effective way to manage the blue cod population* |
| ***Descriptive Social Norm***  “The descriptive norm describes what is typical or *normal.* It is what most people do…” [6, p. 1015] | 1. The majority of recreational fishers normally comply with the daily limit for blue cod* 2. Among recreational fishers I know, most sometimes keep more than the daily limit for blue cod 3. It is common practice among recreational fishers to sometimes keep more than the daily limit for blue cod |
| ***Injunctive Social Norm***  “..rules or beliefs as to what constitutes morally approved and disapproved conduct. Injunctive norms specify what ought to be done” [6, p. 1015] | 1. If I kept more than the daily limit of blue cod my reputation as a fisher would be harmed 2. Doing what other recreational fishers think I should do regarding the daily limit for blue cod is important to me 3. Other recreational fishers would disapprove if I kept more than the daily limit for blue cod 4. People that are important to me, like my friends and family, would not disapprove if I took more than the daily limit for blue cod* |
| ***Guilt***  A ‘‘painful feeling of regret that is aroused when the actor actually causes, anticipates causing, or is associated with an aversive event” [7, p. 20] | 1. I would not feel guilty taking more than the daily limit of blue cod* 2. I would feel guilty for taking more than my fair share if I exceeded the daily limit for blue cod 3. I would have a bad conscience if I exceeded the daily limit of blue cod 4. I would not feel guilty exceeding the daily limit for blue cod since I am not the only one* |
| ***Perceived Behavioural Control***  An individual’s estimate of their ability to perform a specified behavior [8] | 1. It is easy for me to comply with the daily limit for blue cod 2. On a normal fishing trip I would have trouble complying with the daily limit for blue cod* 3. I am confident in my control to not exceed the daily limit for blue cod 4. It is entirely up to me to not go over the daily limit for blue cod (deleted for both daily and size limits) |
| ***Attitude***  An individual's positive or negative evaluation of self-performance of the particular behavior [8] | 1. I do not agree with the daily limit for blue cod 2. My fishing experience is made less enjoyable by complying with the daily limit for blue cod 3. I do not think it is wrong to take more than the daily limit of blue cod* (deleted for size limit) 4. I would not like exceeding the daily limit for blue cod* (deleted for daily limit) |
| ***Moral Norm***  An individual’s personal view on what is right or wrong | 1. Complying with the daily limit for blue cod is the “right thing to do” 2. It is the moral thing to comply with the daily limit for blue cod 3. Regardless of what other fishers think, I do not feel I should comply with the daily limit for blue cod* 4. Following my own values means I feel obligated to not exceed the daily limit for blue cod |

* Reverse-scored item

The first four constructs applied to both the daily and size limits. For the remaining constructs, the items listed below are for the daily limit. The same items were used for the size limit with minimal word changing; mostly just replacing ‘daily limit’ with ‘size limit’.

**References for the Supplementary Material**

1. Nielsen J, Mathiesen C. Important factors influencing rule compliance in fisheries: Lessons from Denmark. Mar Policy. 2003; 27: 409-416. doi:10.1016/S0308-597X(03)00024-1
2. Viteri C, Chávez C. Legitimacy, local participation, and compliance in the Galápagos Marine Reserve. Ocean Coast Manag. 2007; 50: 253-274. [doi:10.1016/j.ocecoaman.2006.05.002](http://dx.doi.org/10.1016/j.ocecoaman.2006.05.002)
3. Bamberg S, Möser G. Twenty years after Hines, Hungerford, and Tomera: A new meta analysis of psycho-social determinants of pro-environmental behaviour. J Environ Psychol. 2007; 27:14-25. doi: 10.1016/j.jenvp.2006.12.002
4. Becker GS. Crime and punishment: An economic approach. In: Essays in the Economics of Crime and Punishment 1974 Jan 1 (pp. 1-54). NBER.
5. Tyler TR. Why People Obey The Law. New Haven, USA and London, UK: Yale University Press; 1990.
6. Cialdini R. Descriptive social norms as underappreciated sources of social control. Psychometrika. 2007; 72: 263-268. doi:10.1007/s11336-006-1560-6
7. Ferguson TJ, Stegge H. Measuring guilt in children. A rose by any other name still has thorns. In: Bybee J, editor. Guilt and children: San Diego, CA: Academic Press; 1998. pp.19-74.
8. Ajzen I. The theory of planned behavior. Organ Behav Hum. 1991 Dec 1; 50(2): 179-211. [doi:10.1016/0749-5978(91)90020-T](http://dx.doi.org/10.1016/0749-5978(91)90020-T)
